# Consequences of exposure to pollutants on respiratory health: From genetic correlations to causal relationships

**DOI:** 10.1371/journal.pone.0277235

**Published:** 2022-11-17

**Authors:** Salvatore D’Antona, Isabella Castiglioni, Danilo Porro, Claudia Cava

**Affiliations:** 1 Institute of Bioimaging and Molecular Physiology, National Research Council, Milan, Italy; 2 Department of Physics, Università degli Studi di Milano-Bicocca, Milan, Italy; 3 NBFC, National Biodiversity Future Center, Palermo, Italy; Clinic for Infectious and tropical diseases, Clinical centre of Serbia, SERBIA

## Abstract

Modern society grew rapidly over the last few decades and this led to an alarming increase in air pollutants and a worsening of the human health, especially in relation to the respiratory system. Indeed, chronic respiratory diseases were the third main cause of death in 2017, with over 3 million of deaths. Furthermore, the pollution has considerable consequences both for burden medical expenses and environmental. However, the mechanisms linking pollutants to the onset of these diseases remain unclear. Thus, in this study we addressed this problem through the United Kingdom BioBank database, analyzing 170 genome-wide association studies (103 related to respiratory diseases and 67 related to pollutants). We analyzed the genetic correlations and causal relationships of these traits, leveraging the summary statistics and bioinformatics packages such as Linkage Disequilibrium Score Regression and Latent Causal Variable. We obtained 158 significant genetic correlations and subsequently we analyzed them through the Latent Causal Variable analysis, obtaining 20 significant causal relationships. The most significant were between "Workplace full of chemicals or other fumes: Sometimes" and “Condition that has ever been diagnosed by a doctor: Asthma” and between “Workplace very dusty: Sometimes” and “Condition that has ever been diagnosed by a doctor: Emphysema or chronic bronchitis”. Finally, we identified single nucleotide polymorphisms independently associated with sveral pollutants to analyze the genes and pathways that could be involved in the onset of the aforementioned respiratory system disorders and that could be useful clinical target. This study highlighted how crucial are the air condition of the working environments and the type of transport used in the onset of respiratory-related morbidity. Based on that, we also suggested some interventions, in order to improve quality life and develop new and eco-friendly society and life style, such as improving indoor air circulation, the use of public transport and urban reforestation.

## Introduction

In 2017, over 544 million people in the world had a chronic respiratory disease, representing an increase of 39.8% compared with 1990, with the highest prevalence among both females and males, in high-income countries [1]. Chronic respiratory diseases were the third main cause of death in 2017, with over 3 million of deaths, behind cardiovascular diseases and cancer [1] and to date, a wide scientific literature acknowledges the primary cause of these respiratory disorders to be pollution [2–5]. Although, the health risks from pollutants such as automobile exhaust gas have been known since 1923 [6], nowadays they still represent one of the most important topics for public opinion and scientific research. During the last century, efforts have been made in order to reduce these pollutants to improve health conditions and the environmental impact [7].

It is possible to categorize air pollutants into two large groups: i) gaseous and ii) particulate (accessed on 02 March 2022) [8, 9]. i) Among gaseous air pollutants there are oxides of nitrogen (NOx). Within the group of NOx, the main one is nitrogen dioxide (NO2), which is produced by the combustion of fossil fuels and by agriculture [10, 11]. ii) Particulate matter (pm) is defined as “a complex mixture with components having diverse chemical and physical characteristics”. It is possible to divide the pm into three sub-categories: particles smaller than 0.1 μm (pm0.1), particles smaller than 2.5 μm (pm2.5) and particles smaller than 10 μm (pm10). Particulate air pollutants are soot, dust and smoke. Sources of pm are, e.g., residual oil and diesel fuel combustion and two-stroke vehicles [8]. pm is primarily emitted from road transportation [12]. However, particular attention should also be paid to chemical compounds that may be present indoors and that if inhaled, pose a threat to an individual’s health (e.g., asbestos, glues and paints) [13, 14]. As a result of the growing concern about the increase of these dangerous air pollutants, the World Health Organization (WHO) has drafted a set of guidelines for countries around the world. These guidelines allow to keep under control the concentrations of these gases and particulates [15] (accessed on 10 January 2022).

To date, the genetic mechanisms shared between being exposed to pollutants and the onset of respiratory diseases are not completely clear and for this reason we investigated the possible relationships between these aspects. Due to the complexity of the genetic architecture of several respiratory diseases we used genome-wide association studies (GWAS), that investigate the connections between millions of single nucleotide polymorphisms (SNPs) present in the whole genome and a wide variety of phenotypes, in order to identify the most significant associations [16]. Specifically, in this study we addressed this challenge using the information present in the database United Kingdom Biobank (UKBB) that include air pollutants and respiratory diseases. This database provides high quality information for millions of SNPs and several phenotypes of interest related to pollutants and respiratory disorders, hence allowing an accurate and comprehensive GWAS that revealed interesting genetic correlations and the causal relationships between the genetic architecture of people exposed to certain pollutants and the onset of respiratory diseases. Finally, once we identified the most statistically significant genetic correlations and causal relationships, we identified the SNPs most independently associated with the aforementioned pollutants (i.e., SNPs index) and, subsequently, the genes and pathways that are involved by these variants. Thus, we identified the most affected genetic traits that could link exposures to air pollution and respiratory diseases.

## Results

After the selection of SNPs based on Minor Allele Frequency (MAF) and minor allele count and after the munge procedure, we calculated the heritability and the z-score of heritability for each of 170 phenotypes relative to respiratory system disorders and air pollutants with the Linkage Disequilibrium Score regression package (LDSC). The overall SNP-based heritability for the pollutant phenotypes ranged from 0.0059 to 0.0443, whereas the corresponding heritabilities for the respiratory disorders ranged from 0.0075 to 0.1758. After selecting phenotypes with z-score heritability > 4, we obtained a list of 43 phenotypes out of 170 of interest (20 phenotypes related to respiratory system and 23 related to pollution). S1 Table provides details of the heritability calculated among the GWAS datasets investigated. The higher the z-score, the more representative are the summary statistics, relatively to their phenotype, based on the quality of their SNPs.

Afterward, we tested these 43 phenotypes through the LDSC package, in order to investigate the genetic correlations between each pollutant and the respiratory disorders. We obtained 158 statistically significant correlations with False Discovery Rate (FDR) < 0.05. These involve 21 pollutants such as: “A gas fire that you use regularly in winter time”, “An open solid fuel fire that you use regularly in winter time”, “Home area population density urban or rural England or Wales: Urban less sparse”, “Nitrogen dioxide air pollution 2010”, “Nitrogen oxides air pollution 2010”, “Particulate matter air pollution pm2 5 2010”, “Time spent driving”, “Transport type for commuting to job workplace: Car or motor vehicle”, “Transport type for commuting to job workplace: Cycle”, “Transport type for commuting to job workplace: Public transport”, “Transport type for commuting to job workplace: Walk”, “Types of transport used (excluding work): Car or motor vehicle”, “Types of transport used (excluding work): Cycle”, “Types of transport used (excluding work): Public transport”, “Types of transport used (excluding work): Walk”, “Worked with materials containing asbestos: Sometimes”, “Worked with paints thinners or glues: Sometimes”, “Workplace full of chemical or other fumes: Sometimes”, “Workplace had a lot of diesel exhaust: Sometimes”, “Workplace very dusty: Often”, “Workplace very dusty: Sometimes” and 20 pathological phenotypes (some of them are classified with the ICD10 codes that refer to the International Statistical Classification of Diseases and Related Health Problems 10th Revision, produced and maintained by the WHO. The aim of ICD-10 is to have a global nomenclature, in order to simplify the systematic recording, analysis, interpretation and comparison of clinical data. https://dhcw.nhs.wales/information-services/information-standards/clinical-classifications-and-terminology-standards/about-clinical-classifications-and-coding/icd-10/). The 20 pathological phenotypes are: “Age asthma diagnosed”, “Age asthma diagnosed by doctor”, “Age hay fever or allergic rhinitis diagnosed by doctor”, “Condition that has ever been diagnosed by a doctor: Asthma”, “Condition that has ever been diagnosed by a doctor: Emphysema or chronic bronchitis”, “Condition that has ever been diagnosed by a doctor: Hay fever allergic rhinitis or eczema”, “Condition that has ever been diagnosed by a doctor: None of the above”, “Breathing problems during period of job”, “Bring up phlegm or sputum or mucus on most days”, “Cough on most days”, “Diagnoses main (ICD10) J44: Other chronic obstructive pulmonary disease”, “Diagnoses main ICD10 R07: Pain in throat and chest”, “Diseases of the respiratory system”, “Doctor diagnosed asthma”, “Doctor diagnosed hay fever or allergic rhinitis”, “Medication related adverse effects (Asthma or COPD)”, “Self-reported: asthma”, “Self-reported: emphysema or chronic bronchitis”, “Self-reported: hay fever or allergic rhinitis”, “Other pulmonary diagnosis”. S2 Table provides details of the genetic correlations calculated among the GWAS datasets investigated.

A positive genetic correlation was obtained between:

- “A gas fire that you use regularly in winter time” with one disorder (“Condition that has ever been diagnosed by a doctor: Emphysema or chronic bronchitis”). It has a genetic correlation = 0.2553, standard error (SE) = 0.1038 and FDR = 0.046.
- “Home area population density urban or rural: England or Wales: Urban less sparse” and 4 respiratory disorders (“Condition that has ever been diagnosed by a doctor: Asthma”, “Diagnoses main ICD10 J44: Other chronic obstructive pulmonary disease”, “Diagnoses main ICD10 R07: Pain in throat and chest”, “Self-reported: asthma”), with genetic correlations from 0.2157, SE = 0.0658 and FDR = 0.005 (“Condition that has ever been diagnosed by a doctor: Asthma”), to 0.3506, SE = 0.1399 and FDR = 0.0422 (“Diagnoses main ICD10 J44: Other chronic obstructive pulmonary disease”).
- “Nitrogen dioxide air pollution 2010” and 1 disorder (“Age hay fever or allergic rhinitis diagnosed by doctor”). It has a genetic correlation = 0.2927, SE = 0.1098 and FDR = 0.0292.
- “Nitrogen oxides air pollution 2010” and 2 pathological phenotypes (“Age hay fever or allergic rhinitis diagnosed by doctor”, “Bring up phlegm or sputum or mucus on most days”) with respectively a genetic correlation = 0.3002, SE = 0.1132 and FDR = 0.0297 and genetic correlation = 0.3288, SE = 0.1033, FDR = 0.007.
- “Particulate matter air pollution (pm2.5) 2010” and 8 respiratory disorders (“Age asthma diagnosed”, “Age hay fever or allergic rhinitis diagnosed by doctor”, “Condition that has ever been diagnosed by a doctor: Asthma’’, “Bring up phlegm or sputum or mucus on most days’’, “Cough on most days’’, “Diagnoses main ICD10 R07: Pain in throat and chest”, “Self-reported: asthma”, “Self-reported: emphysema or chronic bronchitis”) with genetic correlations from 0.134, SE = 0.0493 and FDR = 0.0256 (“Self-reported: asthma”), to 0.4063, SE = 0.1075 and FDR = 0.0012 (“Bring up phlegm or sputum or mucus on most days”).
- “Time spent driving” and 2 pathological phenotypes (“Diseases of the respiratory system”,”Other pulmonary diagnosis”) with genetic correlation = 0.1707, SE = 0.0601 and FDR = 0.018 for both.
- “Transport type for commuting to job workplace: Car or motor vehicle”and 8 respiratory disorders (“Condition that has ever been diagnosed by a doctor: Asthma”, “Condition that has ever been diagnosed by a doctor: Emphysema or chronic bronchitis”, “Diagnoses main ICD10 J44: Other chronic obstructive pulmonary disease”, “Diagnoses main ICD10 R07: Pain in throat and chest”, “Diseases of the respiratory system”, “Self-reported: asthma”, “Self-reported: emphysema or chronic bronchitis”, “Other pulmonary diagnosis”) with genetic correlations from 0.1289, SE = 0.0485, FDR = 0.029625 (“Condition that has ever been diagnosed by a doctor: Asthma”), to 0.4572, SE = 0.1057, FDR = 0.00015 (“Diagnoses main ICD10 J44: Other chronic obstructive pulmonary disease”).
- “Transport type for commuting to job workplace: Public transport” and 3 respiratory disorders (”Age hay fever or allergic rhinitis diagnosed by doctor”,”Condition that has ever been diagnosed by a doctor: Hay fever allergic rhinitis or eczema”,”Self-reported: hay fever or allergic rhinitis”) with genetic correlation from 0.1567, SE = 0.0411, FDR = 0.0006 (“Condition that has ever been diagnosed by a doctor: Hay fever allergic rhinitis or eczema”), to 0.236, SE = 0.0928, FDR = 0.0388 (“Age hay fever or allergic rhinitis diagnosed by doctor”).
- “Transport type for commuting to job workplace: Walk” and 2 pathological phenotypes (“Condition that has ever been diagnosed by a doctor: Hay fever allergic rhinitis or eczema” and “Self-reported: hay fever or allergic rhinitis”) with genetic correlation from 0.217, SE = 0.0546, FDR = 0.0005 (“Condition that has ever been diagnosed by a doctor: Hay fever allergic rhinitis or eczema”), to 0.2714, SE = 0.0785, FDR = 0.002783505 (“Self-reported: hay fever or allergic rhinitis”).
- “Types of transport used (excluding work): Public transport” and 1 pathological phenotype (“Condition that has ever been diagnosed by a doctor: Hay fever allergic rhinitis or eczema”) with genetic correlation = 0.1076, SE = 0.0314 and FDR = 0.0032.
- “Types of transport used (excluding work): Walk” and 2 respiratory disorders (“Condition that has ever been diagnosed by a doctor: Hay fever allergic rhinitis or eczema” and “Self-reported: hay fever or allergic rhinitis”) with genetic correlation from 0.1743, SE = 0.0327, FDR = 2.334273e-06 (“Condition that has ever been diagnosed by a doctor: Hay fever allergic rhinitis or eczema”), to 0.2101, SE = 0.047, FDR = 9.379565e-05 (“Self-reported: hay fever or allergic rhinitis”).
- “Worked with materials containing asbestos: Sometimes” and 7 respiratory disorders (“Condition that has ever been diagnosed by a doctor: Emphysema or chronic bronchitis”, “Cough on most days”, “Diagnoses main ICD10 J44: Other chronic obstructive pulmonary disease”, “Diagnoses main ICD10 R07: Pain in throat and chest”, “Diseases of the respiratory system”, “Self-reported: emphysema or chronic bronchitis”, “Other pulmonary diagnosis”) with genetic correlations from 0.3198, SE = 0.1066 and FDR = 0.0125 (“Cough on most days”), to 0.4586, SE = 0.1405 and FDR = 0.0057 (“Diagnoses main ICD10 J44: Other chronic obstructive pulmonary disease”).
- “Worked with paints thinners or glues: Sometimes” and 10 pathological phenotypes (“Condition that has ever been diagnosed by a doctor: Asthma”, “Condition that has ever been diagnosed by a doctor: Emphysema or chronic bronchitis”, “Bring up phlegm or sputum or mucus on most days”, “Cough on most days”, “Diagnoses main ICD10 J44: Other chronic obstructive pulmonary disease”, “Diagnoses main ICD10 R07: Pain in throat and chest”, “Diseases of the respiratory system”, “Self-reported: asthma”, “Self-reported: emphysema or chronic bronchitis”, “Other pulmonary diagnosis”) with genetic correlations values from 0.1413, SE = 0.0561, FDR = 0.0411 (“Condition that has ever been diagnosed by a doctor: Asthma”), to 0.5807, SE = 0.149, FDR = 0.0006 (“Bring up phlegm or sputum or mucus on most days”).
- “Workplace full of chemical or other fumes: Sometimes” and 11 respiratory disorders (“Condition that has ever been diagnosed by a doctor: Asthma”, “Condition that has ever been diagnosed by a doctor: Emphysema or chronic bronchitis”, “Breathing problems during period of job”, “Bring up phlegm or sputum or mucus on most days”, “Cough on most days”, “Diagnoses main ICD10 J44: Other chronic obstructive pulmonary disease”, “Diagnoses main ICD10 R07: Pain in throat and chest”, “Diseases of the respiratory system”, “Self-reported: asthma”, “Self-reported: emphysema or chronic bronchitis”, “Other pulmonary diagnosis”) with genetic correlations from 0.2111, SE = 0.0608, FDR = 0.0027 (“Self-reported: asthma”), to 0.667, SE = 0.1558, FDR = 0.0001 (“Bring up phlegm or sputum or mucus on most days”).
- “Workplace had a lot of diesel exhaust: Sometimes” and 13 pathological phenotypes (“Condition that has ever been diagnosed by a doctor: Asthma”,”Condition that has ever been diagnosed by a doctor: Emphysema or chronic bronchitis”, “Breathing problems during period of job’’, “Bring up phlegm or sputum or mucus on most days’’, “Cough on most days’’, “Diagnoses main ICD10 J44: Other chronic obstructive pulmonary disease”, “Diagnoses main ICD10 R07: Pain in throat and chest”, “Diseases of the respiratory system”, “Doctor diagnosed asthma”, “Medication related adverse effects (Asthma or COPD)”, “Self-reported: asthma”, “Self-reported: emphysema or chronic bronchitis”, “Other pulmonary diagnosis” with genetic correlations from 0.1797, SE = 0.0659, FDR = 0.025 (“Self-reported: asthma”), to 0.5988, SE = 0.1464, FDR = 0.0003 (“Bring up phlegm or sputum or mucus on most days”).
- “Workplace very dusty: Often” and 13 pathological phenotypes (“Age asthma diagnosed”, “Condition that has ever been diagnosed by a doctor: Asthma”, “Condition that has ever been diagnosed by a doctor: Emphysema or chronic bronchitis”, “Breathing problems during period of job”, “Bring up phlegm or sputum or mucus on most days”, “Cough on most days”, “Diagnoses main ICD10 J44: Other chronic obstructive pulmonary disease”, “Diagnoses main ICD10 R07: Pain in throat and chest”, “Diseases of the respiratory system”, “Doctor diagnosed asthma”, “Self-reported: asthma”, “Self-reported: emphysema or chronic bronchitis”, “Other pulmonary diagnosis” with genetic correlations from 0.1627, SE = 0.0548 and FDR = 0.0137 (“Condition that has ever been diagnosed by a doctor: Asthma”), to 0.6129, SE = 0.1081, FDR = 4.542e-07 (“Diagnoses main ICD10 J44: Other chronic obstructive pulmonary disease”).
- “Workplace very dusty: Sometimes” and 13 respiratory disorders (“Condition that has ever been diagnosed by a doctor: Asthma”, “Condition that has ever been diagnosed by a doctor: Emphysema or chronic bronchitis”, “Breathing problems during period of job”, “Bring up phlegm or sputum or mucus on most days”, “Cough on most days”, “Diagnoses main ICD10 J44: Other chronic obstructive pulmonary disease”, “Diagnoses main ICD10 R07: Pain in throat and chest”, “Diseases of the respiratory system”, “Doctor diagnosed asthma”, “Medication related adverse effects (Asthma or COPD)”, “Self-reported: asthma”, “Self-reported: emphysema or chronic bronchitis”, “Other pulmonary diagnosis”) with genetic correlation from 0.2855, SE = 0.0783, FDR = 0.0018 (“Self-reported: asthma”), to 0.7579, SE = 0.1803, FDR = 0.0002 (“Bring up phlegm or sputum or mucus on most days”).

We found a negative genetic correlation between:

- “An open solid fuel fire that you use regularly in winter time” and 10 pathological phenotypes (“Condition that has ever been diagnosed by a doctor: Asthma”, “Condition that has ever been diagnosed by a doctor: Emphysema or chronic bronchitis”, “Breathing problems during period of job”, “Cough on most days”, “Diagnoses main ICD10 J44: Other chronic obstructive pulmonary disease”, “Diagnoses main ICD10 R07: Pain in throat and chest”, “Diseases of the respiratory system”, “Self-reported: asthma”, “Self-reported: emphysema or chronic bronchitis”, “Other pulmonary diagnosis”) with genetic correlations from -0.4312, SE = 0.09 and FDR = 2.173e-05 (“Diagnoses main ICD10 J44: Other chronic obstructive pulmonary disease”), to -0.1701, SE = 0.0429, FDR = 0.0005 (“Self-reported: asthma”).
- “Time spent driving” and 1 inverse correlation (”Self-reported: hay fever or allergic rhinitis”). It has a genetic correlation = -0.1367, SE = 0.0427 and FDR = 0.007
- “Transport type for commuting to job workplace: Car or motor vehicle”and 2 inverse correlation (“Age hay fever or allergic rhinitis diagnosed by doctor” and”Self-reported: hay fever or allergic rhinitis”) with respectively genetic correlation = -0.2791, SE = 0.1045, FDR = 0.029 (“Age hay fever or allergic rhinitis diagnosed by doctor”) and genetic correlation = -0.1807, SE = 0.0621, and FDR = 0.015.
- “Transport type for commuting to job workplace: Cycle” and 10 respiratory disorders (“Condition that has ever been diagnosed by a doctor: Asthma”, “Condition that has ever been diagnosed by a doctor: Emphysema or chronic bronchitis”, “Breathing problems during period of job”, “Cough on most days”, “Diagnoses main ICD10 J44: Other chronic obstructive pulmonary disease”, “Diagnoses main ICD10 R07: Pain in throat and chest”, “Diseases of the respiratory system”, “Self-reported: asthma”, “Self-reported: emphysema or chronic bronchitis”, “Other pulmonary diagnosis”) with genetic correlations from -0.4823, SE = 0.0608, FDR = 5.724e-13 (“Diagnoses main ICD10 R07: Pain in throat and chest”), to -0.1094, SE = 0.0415, FDR = 0.031 (“Self-reported: asthma”).
- “Transport type for commuting to job workplace: Public transport” and 6 respiratory disorders (“Condition that has ever been diagnosed by a doctor: Emphysema or chronic bronchitis”, “Diagnoses main ICD10 J44: Other chronic obstructive pulmonary disease”, “Diagnoses main ICD10 R07: Pain in throat and chest”, “Diseases of the respiratory system”, “Self-reported: emphysema or chronic bronchitis”, “Other pulmonary diagnosis”) with genetic correlations from -0.5054, SE = 0.0746 and FDR = 1.107e-09 (“Diseases of the respiratory system”, “Other pulmonary diagnosis”), to -0.2801, SE = 0.0886 and FDR = 0.0079 (“Self-reported: emphysema or chronic bronchitis”).
- “Transport type for commuting to job workplace: Walk” and 5 respiratory disorders (“Condition that has ever been diagnosed by a doctor: Emphysema or chronic bronchitis”,”Diagnoses main ICD10 J44: Other chronic obstructive pulmonary disease”,”Diagnoses main ICD10 R07: Pain in throat and chest”,”Diseases of the respiratory system”,”Other pulmonary diagnosis”) with genetic correlations values from -0.3907, SE = 0.125 and FDR = 0.0087 (“Diagnoses main ICD10 J44: Other chronic obstructive pulmonary disease”), to -0.2472, SE = 0.0853, FDR = 0.01628571 (“Diagnoses main ICD10 R07: Pain in throat and chest”).
- “Types of transport used (excluding work): Car or motor vehicle” and 1 pathological phenotype (“Self-reported: emphysema or chronic bronchitis”) with a genetic correlation = -0.1992, SE = 0.0654, FDR = 0.0109.
- “Types of transport used (excluding work): Cycle” and 10 pathological phenotypes (“Condition that has ever been diagnosed by a doctor: Asthma”, “Condition that has ever been diagnosed by a doctor: Emphysema or chronic bronchitis”, “Cough on most days”, “Diagnoses main ICD10 J44: Other chronic obstructive pulmonary disease”, “Diagnoses main ICD10 R07: Pain in throat and chest”, “Diseases of the respiratory system”, “Medication related adverse effects (Asthma or COPD)”, “Self-reported: asthma”, “Self-reported: emphysema or chronic bronchitis”, “Other pulmonary diagnosis” with genetic correlations from -0.4286, SE = 0.0498, FDR = 4.36e-15 (“Diagnoses main ICD10 R07: Pain in throat and chest”), to -0.1296, SE = 0.0393 and FDR = 0.0052 (“Self-reported: asthma”).
- “Types of transport used (excluding work): Public transport” and 3 respiratory disorders (”Diagnoses main ICD10 R07: Pain in throat and chest”,”Diseases of the respiratory system”,”Other pulmonary diagnosis”) with genetic correlations values from -0.2324, SE = 0.0658 and FDR = 0.0023 (“Diseases of the respiratory system” and “Other pulmonary diagnosis”), to -0.1671, SE = 0.0483, FDR = 0.0027 (“Diagnoses main ICD10 R07: Pain in throat and chest”).
- “Types of transport used (excluding work): Walk” and 8 pathological phenotypes (“Condition that has ever been diagnosed by a doctor: Asthma”, “Condition that has ever been diagnosed by a doctor: Hay fever allergic rhinitis or eczema”,”Diagnoses main ICD10 J44: Other chronic obstructive pulmonary disease”,”Diagnoses main ICD10 R07: Pain in throat and chest”,”Diseases of the respiratory system”,”Self-reported: asthma”,”Self-reported: emphysema or chronic bronchitis”,”Other pulmonary diagnosis”) with genetic correlation from -0.3274, SE = 0.046 and FDR = 1.498e-10 (“Diagnoses main ICD10 R07: Pain in throat and chest”), to -0.0853, SE = 0.0332, FDR = 0.036 (“Self-reported: asthma”).
- “Workplace very dusty: Sometimes” and 1 pathological phenotype (“Condition that has ever been diagnosed by a doctor: None of the above”) with genetic correlation = -0.1614, SE = 0.0647 and FDR = 0.0427.

**Fig 1:**
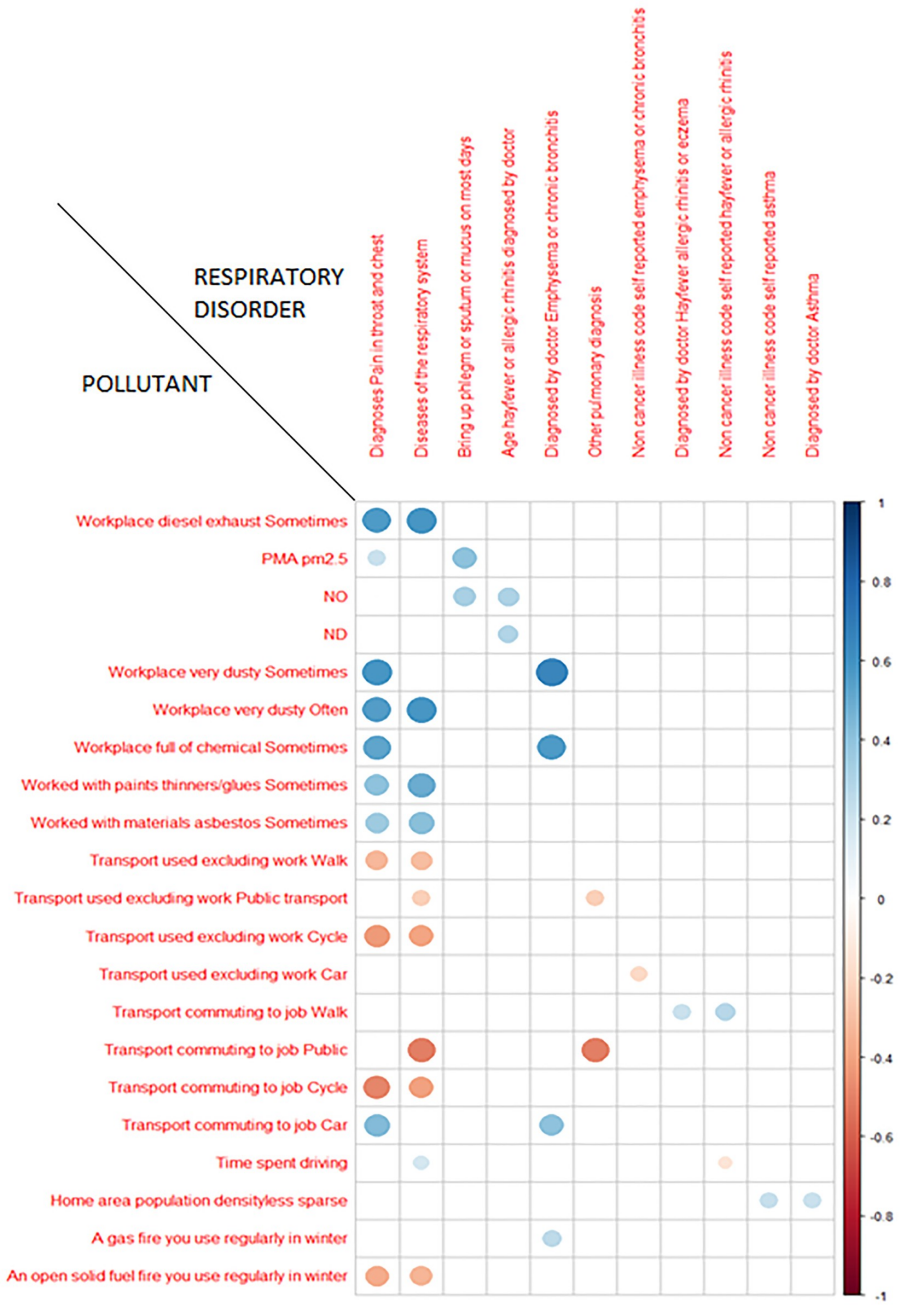
Top genetic correlations. Chart reports the most significant Genetic Correlations for each pollutant. The size and color of the dots represent respectively the genetic correlation measurement. The blue color represents a positive correlation, while the orange color represents a negative correlation. NO: Nitrogen oxides air pollution; 2010. ND: Nitrogen dioxide air pollution; 2010. PMA pm2.5: Particular matter air with diameter less than 2.5 mm.

Below (Fig 1) we briefly report these genetic correlations, values showing the top 2 correlations for each pollutant, based on the lowest FDR.

Based on these results, subsequently we selected the 158 summary statistics of the phenotypes with the most significant associations, and we submitted them to the Latent Causal Variable (LCV) package, in order to test whether the significant genetic correlation results were due to causal effects rather than shared genetic mechanisms. After the FDR correction, we observed that 20 causal relationships were statistically significant (Table 1).

**Table 1:** 20 significant causal relationships between air pollutants and respiratory disorders.

**An open solid fuel fire that you use regularly in winter time**		
	**Estimated posterior gcp (SE)**	**FDR**
Condition that has ever been diagnosed by a doctor: Asthma	-0.63 (0.24)	0.0264
Self-reported: asthma	-0.63 (0.24)	0.0221
**Transport type for commuting to job workplace: Car or motor vehicle**		
	**Estimated posterior gcp (SE)**	**FDR**
Age hay fever or allergic rhinitis diagnosed by doctor	-0.30 (0.19)	0.0148
Diseases of the respiratory system	0.77 (0.30)	7.03e-07
Other pulmonary diagnosis	0.77 (0.30)	7.03e-07
**Transport type for commuting to job workplace: Public transport**		
	**Estimated posterior gcp (SE)**	**FDR**
Age hay fever or allergic rhinitis diagnosed by doctor	-0.68 (0.18)	2.86e-08
Self-reported: emphysema or chronic bronchitis	-0.61 (0.24)	0.009
**Transport type for commuting to job workplace: Walk**		
	**Estimated posterior gcp (SE)**	**FDR**
Self-reported: hay fever or allergic rhinitis	0.56 (0.25)	4.00e-12
**Types of transport used (excluding work): Car or motor vehicle**		
	**Estimated posterior gcp (SE)**	**FDR**
Self-reported: emphysema or chronic bronchitis	-0.38 (0.15)	0.031
**Worked with materials containing asbestos: Sometimes**		
	**Estimated posterior gcp (SE)**	**FDR**
Cough on most days	0.66 (0.39)	0.0001
**Worked with paints thinners or glues: Sometimes**		
	**Estimated posterior gcp (SE)**	**FDR**
Bring up phlegm or sputum or mucus on most days	0.11 (0.82)	1.05e-08
**Workplace full of chemical or other fumes: Sometimes**		
	**Estimated posterior gcp (SE)**	**FDR**
Condition that has ever been diagnosed by a doctor: Asthma	0.48 (0.15)	3.16e-17
Bring up phlegm or sputum or mucus on most days	-0.52 (0.77)	1.59e-17
Self-reported: asthma	0.50 (0.13)	1.99e-16
**Workplace very dusty: Sometimes**		
	**Estimated posterior gcp (SE)**	**FDR**
Condition that has ever been diagnosed by a doctor: Emphysema or chronic bronchitis	0.82 (0.14)	6.33e-05
Breathing problems during period of job	-0.36 (0.28)	0.0084
Cough on most days	-0.61 (0.54)	1.99e-16
Diagnoses main ICD10 R07: Pain in throat and chest	0.79 (0.18)	0.0014
Diseases of the respiratory system	0.81 (0.17)	0.0009
Other pulmonary diagnosis	0.81 (0.17)	0.0009

gcp: genetic causal proportion; SE: standard error; FDR: false discovery rate.

We calculated genetic causality proportion (gcp) for each air pollutant and respiratory disorder.

“Transport type for commuting to job workplace: Car or motor vehicle” had a nearly-full genetic causality (gpc > 0.60) with the onset of “Diseases of the respiratory system” and “Other pulmonary diagnosis”, with gcp = 0.77 and FDR = 7.03e-07 for both.

The pollutant “Transport type for commuting to job workplace: Walk” has partial (gcp < 0.60), but significant genetic causality in the onset of “Self-reported: hay fever or allergic rhinitis”, with gcp = 0.56 and FDR = 4.00e-12.

The pollutant “Worked with materials containing asbestos: Sometimes” showed a nearly-full genetic causality with the onset of “Cough on most days”, with a gcp = 0.66 and FDR = 0.0001.

We observed that some pollutant phenotypes such as "Workplace full of chemicals or other fumes: Sometimes" have partial (gcp < 0.60), but significant genetic causality in the onset of some respiratory disorders (e.g., “Condition that has ever been diagnosed by a doctor: Asthma” gcp = 0.48, FDR = 3.16e-17, and “Self-reported: asthma”, gcp = 0.50, FDR = 1.99e-16).

A nearly-full genetic causality was found between “Workplace very dusty: Sometimes” and in the onset of some respiratory disorders (e.g., “Condition that has ever been diagnosed by a doctor: Emphysema or chronic bronchitis”, gcp = 0.82, FDR = 6.33e-05, and “Diseases of the respiratory system” gcp = 0.81, FDR = 0.0009, and “Other pulmonary diagnosis” gcp = 0.81, FDR = 0.0009). Our results did not identify causal relationships between pollutants such as “Nitrogen dioxide air pollution 2010”, “Nitrogen oxides air pollution 2010” and “Particulate matter air pollution (pm2.5) 2010” and the onset of respiratory complications.

Afterward, in order to investigate the genetic traits involved in the complex interactions between these phenotypes, we selected the summary statistics of 3 pollutants with a significant causal relationship (i.e., “Transport type for commuting to job workplace: Car or motor vehicle”, “Workplace full of chemicals or other fumes: Sometimes” and “Workplace very dusty: Sometimes”) and we identified through PLINK software the SNPs index of each polluting agent. Subsequently, we submitted the list of SNPs index to Biomart and EnsDb.Hsapiens.v79, thus we could identify the variants and the genes that were being affected. As expected, most of the variants identified are related to intronic regions. For the pollutants “Transport type for commuting to job workplace: Car or motor vehicle”, “Workplace full of chemicals or other fumes: Sometimes” and “Workplace very dusty: Sometimes” we identified 23, 9 and 7 genes, respectively that we show below (Table 2).

**Table 2:** SNP index, their types and the genes they affect, related to pollutants with the top 3 most statistically significant causal relationships.

**Transport type for commuting to job workplace: Car or motor vehicle**		
***SNP***	***Consequence type***	***Gene***
rs10188046	intron variant	*TSSC1*
rs10968219	3 prime UTR variant	*LINGO2*
rs112816036	intron variant	*KDM4C*
rs11759065	intron variant	*PPP1R14C*
rs12127101	intron variant	*DMBX1*
rs12410877	intron variant	*HHAT*
rs12555384	intron variant	*UNC13B*
rs13145199	intron variant	*FSTL5*
rs1370216	intron variant	*WDR7*
rs1381109	intron variant	*SCN1A*
rs1382635	intron variant	*NAV3*
rs142472919	intron variant	*CPT1C*
rs1641843	synonymous variant	*SNX29*
rs17415991	intron variant	*DNAJC16*
rs366309	intron variant	*COA7*
rs4683131	intron variant	*LIMD1*
rs4753063	intron variant	*FAT3*
rs5014494	intron variant	*SYCP2L*
rs56003481	intron variant	*LPIN3*
rs75061507	intron variant	*KCND3*
rs76264086	intron variant	*SYNE1*
rs7743791	intron variant	*PARK2*
rs7893285	intron variant	*MICU1*
**Workplace full of chemical or other fumes: Sometimes**		
***SNP***	***Consequence type***	***Gene***
rs10834475	Intron variant	*LUZP2*
rs111777044	Intron variant	*CDKAL1*
rs113361079	intron variant	*ABCA13*
rs138621393	3 prime UTR variant	*DOCK3*
rs147158641	intron variant	*PPFIBP1*
rs1665689	intron variant	*CDH23*
rs67413306	intron variant	*RAB4A*
rs9750811	intron variant	*PARD3B*
rs9836755	intron variant	*CADM2*
rs10834475	intron variant	*LUZP2*
**Workplace very dusty: Sometimes**		
***SNP***	***Consequence type***	***Gene***
rs10456790	intron variant	*ATXN1*
rs115051693	intron variant	*REV1*
rs118077376	intron variant	*TPD52*
rs2715686	intron variant	*GUCA1C*
rs4102412	intron variant	*ZNF385D*
rs567171971	intron variant	*FBXO8*
rs76586583	intron variant	*CARS2*

SNP: single nucleotide polymorphism.

Finally, we submitted the list of genes to Reactome software, in order to identify pathways involved and that could be clinical targets. Therefore, we obtained a list of pathways that we briefly report below (Table 3), indicating for each gene the pathway involved. For “Transport type for commuting to job workplace: Car or motor vehicle”, “Workplace full of chemicals or other fumes: Sometimes” and “Workplace very dusty: Sometimes” we identified 17, 14 and 12 pathways.

**Table 3:** The most significant pathways enriched by the genes associated with pollutants. The table shows the pathway name, P value and the genes of our list that are related to that pathway.

**Transport type for commuting to job workplace: Car or motor vehicle**		
***Pathway***	***P value***	***Genes found***
HHAT G278V doesn’t palmitoylate Hh-Np	0.008	*HHAT*
Phase 1—inactivation of fast Na+ channels	0.014	*KCND3*
Nef Mediated CD4 Down-regulation	0.020	*PPP1R14C*
Josephin domain DUBs	0.023	*PARK2*
Synthesis of PE	0.025	*LPIN3*
Cardiac conduction	0.027	*KCND3;SCN1A*
Triglyceride biosynthesis	0.027	*LPIN3*
Depolymerisation of the Nuclear Lamina	0.031	*LPIN3*
Processing of SMDT1	0.031	*MICU1*
Acetylcholine Neurotransmitter Release Cycle	0.033	*UNC13B*
Serotonin Neurotransmitter Release Cycle	0.035	*UNC13B*
Norepinephrine Neurotransmitter Release Cycle	0.035	*UNC13B*
Synthesis of PIPs at the Golgi membrane	0.035	*PPP1R14C*
PINK1-PRKN Mediated Mitophagy	0.043	*PARK2*
Nef-mediates down modulation of cell surface receptors by recruiting them to clathrin adapters	0.043	*PPP1R14C*
Dopamine Neurotransmitter Release Cycle	0.045	*UNC13B*
Mitochondrial calcium ion transport	0.045	*MICU1*
Glutamate Neurotransmitter Release Cycle	0.046	*UNC13B*
**Workplace full of chemical or other fumes: Sometimes**		
***Pathway***	***P value***	***Genes found***
NTRK2 activates RAC1	0.004	*DOCK3*
Activated NTRK2 signals through FYN	0.005	*DOCK3*
MET receptor recycling	0.008	*RAB4A*
Receptor-type tyrosine-protein phosphatases	0.015	*PPFIBP1*
Signaling by NTRK2 (TRKB)	0.021	*DOCK3*
Adherens junctions interactions	0.025	*CADM2*
tRNA modification in the nucleus and cytosol	0.033	*CDKAL1*
TBC/RABGAPs	0.034	*RAB4A*
Synthesis of PIPs at the plasma membrane	0.040	*RAB4A*
Sensory processing of sound by outer hair cells of the cochlea	0.043	*CDH23*
Signaling by ALK fusions and activated point mutants	0.043	*PPFIBP1*
Signaling by ALK in cancer	0.043	*PPFIBP1*
RAB geranylgeranylation	0.049	*RAB4A*
Cell-cell junction organization	0.050	*CADM2*
**Workplace very dusty: Sometimes**		
***Pathway***	***P value***	***Genes found***
Translesion synthesis by REV1	0.010	*REV1*
Translesion synthesis by POLI	0.010	*REV1*
Translesion synthesis by POLK	0.010	*REV1*
Mitochondrial tRNA aminoacylation	0.013	*CARS2*
Termination of translesion DNA synthesis	0.019	*REV1*
Inactivation	0.003	-
The phototransduction cascade	0.020	*GUCA1C*
Translesion synthesis by Y family DNA polymerases bypasses lesions on DNA template	0.023	*REV1*
tRNA Aminoacylation	0.025	*CARS2*
DNA Damage Bypass	0.029	*REV1*
Golgi Associated Vesicle Biogenesis	0.033	*TPD52*
trans-Golgi Network Vesicle Budding	0.044	*TPD52*

For “Transport type for commuting to job workplace: Car or motor vehicle” (that has a causal effect on “Diseases of the respiratory system” and “Other pulmonary diagnosis”) the most statistically significant pathway is the one involving "HHAT G278V non-palmitoyl Hh-Np," p value = 0.008, gene involved: hedgehog acyltransferase (*HHAT*, related to rs12410877, an intronic variant [17].

Related to "Workplace full of chemicals or other fumes: Sometimes" (with a causal effect on “Condition that has ever been diagnosed by a doctor: Asthma”) the most statistically significant pathway involved is “NTRK2 activates RAC1”, p value = 0.004, gene involved: dedicator of cytokinesis 3 (*DOCK3*, related to rs138621393, a 3 prime UTR variant) [18].

Regarding “Workplace very dusty: Sometimes” (that has a causal effect on “Condition that has ever been diagnosed by a doctor: Emphysema or chronic bronchitis”) the most statistically significant pathway is “Translation synthesis by REV1”, p value = 0.010, gene involved: REV1 DNA directed polymerase (*REV1*, related to rs115051693, an intronic variant) [19].

## Discussion

Previous GWAS, and observational studies, often use longitudinal approaches to assess the effects of pollutants on participants’ health, collecting questionnaires and medical assessments data and then cross-checking them with parameters like the annual community-level average of pollutants such as: ozone, nitrogen dioxide, and particulate matter air pollution (pm10), or particulate matter air pollution (pm2.5), over the period under study and in the geographic area of interest. Afterward, the results are further corroborated by statistical analyses, such as multilevel Poisson regression model, or Hierarchical multivariate regression model [20–22].

However, one of the limitations of these studies is that the researchers often focus on investigating correlations that exist between the traits examined, or between the phenotypes of interest and the presence of specific SNPs, but not on causal effects. Through a computational approach, we addressed this topic, leveraging the summary statistics present in UKBB related to air pollutants and respiratory disorders and thus investigating the genetic correlations, the underlying causal relationships leading to the onset of these disorders and finally the genetic traits such as the most involved genes and pathways. The results obtained from the LDSC analysis in the present study are confirmed by the literature in relation to low health quality due to life in polluted environments [23]. Indeed, as we expected, we found strong positive genetic correlations between air pollutants such as “Workplace diesel exhaust: Sometimes”, “Workplace very dusty: Often”, “Worked with paints thinners/glues: Sometimes”, “Worked with materials asbestos: Sometimes” and pathological phenotypes, such as “Diagnoses Pain in throat and chest” and “Disease of the respiratory system” [12, 24]. Whereas, as expected, a healthy lifestyle achieved by behaviors such as “Types of transport used (excluding work): Walk”, and “Transport used (excluding work): Cycle”, showed a negative genetic correlation with some respiratory diseases. Probably, this could be due to avoidance of the most polluted roads [25] and this hypothesis could be confirmed analyzing the phenotype "Transport commuting to job: Walk" which instead, involving polluted roads, shows a positive correlation with pathological phenotypes such as " Condition that has ever been diagnosed by a doctor: Hay fever allergic rhinitis or eczema” and “Self-reported: hay fever or allergic rhinitis” (Fig 1). Interestingly, similar exposures such as "A gas fire that is regularly used in winter" and "An open solid fuel fire that is regularly used in winter" are differently correlated with respiratory diseases. Indeed, both share a relationship with one disorder (i.e., "Condition that has been diagnosed by a physician: Emphysema or chronic bronchitis"), but only "A gas fire that is regularly used in winter" showed a positive genetic correlation. Since the literature reports that usually a solid fuel tends to produce worse air quality than a gaseous fuel [26], we believe that the indoor environment in this case has a central role in the human health and therefore it may be healthier if it is open and possibly ventilated. This could additionally explain why "An open solid fuel fire that is regularly used in winter" has been shown to have several negative genetic correlations with additional respiratory tract disorders such as: “Condition that has ever been diagnosed by a doctor: Asthma”, “Breathing problems during period of job”, “Cough on most days”, “Diagnoses main ICD10 J44: Other chronic obstructive pulmonary disease”, “Diagnoses main ICD10 R07: Pain in throat and chest”, “Diseases of the respiratory system”, “Self-reported: asthma”, “Self-reported: emphysema or chronic bronchitis”, “Other pulmonary diagnosis”.

Interestingly, our results suggest that the pollutants most widely recognized as being associated with the onset and exacerbation of respiratory diseases (e.g., particulate air pollution (pm2.5)) do not seem to have a causal effect on the aforementioned diseases. Indeed, our LCV findings show that “Transport type for commuting to job workplace: Car or motor vehicle” and the conditions of the work place (e.g., “Workplace full of chemical or other fumes: Sometimes”, and “Workplace very dusty: Sometimes”) seem to be the main contributors to the onset of several disorders, such as “Diseases of the respiratory system”, “Other pulmonary diagnosis”, “Condition that has ever been diagnosed by a doctor: Asthma”, “Self-reported: asthma“, “Condition that has ever been diagnosed by a doctor: Emphysema or chronic bronchitis”, “Diagnoses main ICD10 R07: Pain in throat and chest”, respectively (Table 1).

Automobile exhaust is known to contain carcinogens that can lead to respiratory disorders, such as lung cancer [27]. Therefore, our results related to the causal effect of “Transport type for commuting to job workplace: Car or motor vehicle” exposure on “Diseases of the respiratory system” and “Other pulmonary diagnosis” conditions are confirmed by literature. Interestingly, this kind of relationship is not present for public transportation, probably due to the fact that in recent decades urban public administrations have been motivated to develop environmentally friendly or electricity-powered connection networks, such as buses and subways.

Among the causality effects, the ones between "Workplace full of chemicals or other fumes: Sometimes" and “Condition that has ever been diagnosed by a doctor: Asthma” and between “Workplace very dusty: Sometimes” and “Condition that has ever been diagnosed by a doctor: Emphysema or chronic bronchitis” are the most statistically significant. These results are expected, indeed previously the National Institute for Occupational Safety and Health reported that between 2011 and 2016 nearly 17% of all adult onset asthma cases were related to the workplace. In addition, the median prevalence of work-exacerbated asthma was 22%, but some studies have suggested that this could be 58% [28]. Furthermore, using the self-reported workplace information collected in the National Health and Nutrition Examination Survey, Doney et al suggested a causal effect of the exposure of dust in the workplace on the onset of emphysema and chronic bronchitis in the employees of industry companies [29].

Our results showed that the most statistically significant pathway related to “Transport type for commuting to job workplace: Car or motor vehicle” (with causal effect on “Diseases of the respiratory system” and “Other pulmonary diagnosis”) is "HHAT G278V non-palmitoyl Hh-Np," (i.e., the Sonic Hedgehog (SHH) morphogen pathway). This is known to have a central role in the embryonic development and stem cell preservation [17]. An important step in this signaling is the transfer of a palmitate group to the SHH N terminus, catalyzed by the multi-pass transmembrane enzyme *HHAT*. However, the involvement of this pathway is expected, since its aberrations are related to several cancers [17]. Indeed, this gene has a ubiquitous expression, including in the lungs. Specifically, the literature reports that *HHAT* overexpression is often found in many tumors (e.g., in lung squamous cells carcinoma) [30]. Therefore, it is likely that a mutation in this gene could lead to the onset of several other respiratory system disorders. Concerning "Workplace full of chemicals or other fumes: Sometimes" (with causal effect on “Condition that has ever been diagnosed by a doctor: Asthma”) the most statistically significant pathway involved is “NTRK2 activates RAC1”. The gene involved is *DOCK3* that mediates the activation of Rac family small GTPase 1 (RAC1) downstream of BDNF-induced signaling by neurotrophic receptor tyrosine kinase 2 (NTRK2) [18]. This pathway has a central role in axonal growth and regeneration [18]. Specifically, *DOCK3* has been shown to be involved in regulation of cytoskeletal organization, cell–cell interactions, and function as a guanine nucleotide exchange factor [31, 32]. *DOCK3* is usually expressed in the brain. Although a previous study suggested that *DOCK3* could have a role in lung cancer [33], to date we are not aware of its implication in asthma. Future studies could validate our finding to obtain a novel target gene in the treatment of asthma. Regarding “Workplace very dusty: Sometimes” (that has a causal effect on “Condition that has ever been diagnosed by a doctor: Emphysema or chronic bronchitis”) the most statistically significant pathway is “Translation synthesis by REV1”, involving the *REV1* gene. The role of this gene is unclear in human. However, it encodes a protein with similarity to the S. cerevisiae protein Rev1, which has a key role in protein-protein interactions. It is assumed that in humans the Rev1-like protein acts as a recruiter for DNA polymerases involved in the synthesis of damaged DNA transions [19]. This gene has a ubiquitous expression, including in the lungs [19]. Although to date we are not aware of any literature describing an association between mutations in this gene and emphysema or chronic bronchitis, a previous study suggest that a reduced concentration of *REV1* transcripts was related with a significant decrease in the diversity of carcinogen-induced lung cancers and complete suppression of tumor development in 27% of the carcinogen-exposed mice models [34]. Therefore, it highlights the key role of the translesion synthesis pathway in the development of lung cancer. Consequently, we suggest that the role of mutations in *REV1* should be investigated more, related to respiratory disorders, in order to better understand its role and to obtain an efficient clinical target.

Accordingly, more focused efforts should be made to improve work and home environment air quality and the type of vehicle used to get to work. Based on this, the problem of pollution has to be addressed at indoor and outdoor level.

At indoor level, in order to improve people’s health conditions, we suggest increasing the ventilation rate in the work places and houses. Indeed, previous studies have shown how an improvement in indoor air given by better ventilation increases productivity and prevents pathological conditions such as rhinitis and the risk of allergic symptoms [24, 35, 36].

At outdoor level, we suggest using public transport to commuting to work, since we did not find a direct causal relationship between this kind of transport and the onset of respiratory diseases. In addition, in order to reduce the production of outdoor pollutants and prevent workers from driving on particularly polluted roads, future efforts could be aimed at encouraging the use of electric or low-emission cars.

In line with this scenario, our genetic correlation analysis showed a positive correlation between “Home area population density urban or rural England or Wales: Urban less sparse” and 4 respiratory disorders, suggesting urban reforestation (i.e., strategies aimed at creating a network or system that includes forests, groups of trees, and individual trees in urban and peri-urban areas, https://www.fao.org/forestry/urbanforestry/en/), to reduce pollutants concentration.

Moreover, as “Home area population density urban or rural England or Wales: Urban less sparse”, although a positive genetic correlation was found between nitrogen dioxide, nitrogen oxides, and particulate air pollution (pm2.5) and several respiratory disorders, genetic causality analysis did not yield the same result. Consequently, it is assumed that the relationship between these pollutants and the occurrence of respiratory disorders is due to shared genetic mechanisms rather than a causal relationship [37, 38]. Furthermore, our results suggest that the genes and pathways that previous studies indicate as being related to the onset of lung cancer could also lead to the development of other respiratory diseases such as asthma or chronic bronchitis. Consequently, although the genetic traits identified in this work need further studies to be validated, they could be interesting clinical targets for the treatment of various respiratory system disorders.

In conclusion, new strategies should be considered to prevent the onset of respiratory disorders and ease the burden of medical expenses that have increased in recent years precisely because of the increased concentration of pollutants [1]. Although in recent decades there has been an increase in awareness of the problems of pollution produced by city life, industries and agriculture, significant challenges remain for society. Among these, the ability to develop eco-friendly cities and lifestyles capable of maintaining not only high production standards, but also and above all a high quality of life. For these purposes, further studies of the impact on the environment and people’s health are needed.

## Materials and methods

### SNPs selection and quality control of summary statistics

Our analyses started with data selection from the UKBB (http://www.nealelab.is/uk-biobank/, accessed on 4 February 2022). This database provides data for over 13 million SNPs, referring to a cohort of approximately 500000 participants of English European descent, aged between 40 and 69 (54% females) [20]. For this study we selected summary statistics that include both sexes, analyzing 170 phenotypes: 103 pathogenic phenotypes related to respiratory system (e.g., “Cough on most days” and “Age asthma diagnosed”) and 67 phenotypes related to pollution factors (e.g., “Nitrogen oxides air pollution; 2010” and “Particulate matter air pollution (pm2.5); 2010”). S3 Table provides the complete list of the phenotypes examined in this work. Once these datasets were obtained, we performed a quality control. Specifically, we selected only the SNPs with a MAF > 0.01 and we removed SNPs with a minor allele count < 20.

### Heritability and genetic correlation analysis

Subsequently, we submitted our data to the LDSC package (version 1.0.1). This package is a tool that refers to GWAS summary statistics for estimating heritability, namely the proportion of phenotypic variance attributable to variance in genetic factors [39], and genetic correlation, a quantitative genetic parameter that describes the rate and relationship between two traits. This should reflect pleiotropic action of genes or correlation between causal loci in two traits [40]. Specifically, in the first step we used the command munge provided by the LDSC package, as is strongly recommend by the developers, in order to convert summary statistics into a format compatible to the package, thus avoiding computational issues. Indeed, the munge procedure eliminates variants that are not SNPs (e.g., indels), duplicated SNPs, strand ambiguous SNPs and with an insufficient sample size. In addition, munge procedure checks that the median value of the signed summary statistic column (e.g., beta) is close to the null median, with the purpose of make sure that this column is not mislabeled [40]. Subsequently, we calculated the heritability and the z-score of heritability for each phenotype. As suggested by the developers, we selected only the significant phenotypes (i.e., with a z-score > 4 [40]). z-score is defined as the ratio between per-allele effect sizes and their standard errors [41] and in general, the scale of the heritability z-score derives by three features: proportion of causal variants, sample size and SNP-based heritability. An increase in these three features cause an increase in the heritability z-score. This indicates that the heritability z-score acquires information about the genetic architecture of traits that have sufficient sample size, high heritability and a high proportion of causal variants [42].

Afterward, we performed the genetic correlation analysis between the selected phenotypes related to the pollution and the selected traits related to the respiratory diseases. This is a method that uses the linkage disequilibrium (LD) mechanism through the genome to estimate the distribution of effect sizes for each SNP as a function of their LD score, thus identifying the score and the kind of correlations between phenotypes. For the purpose of selecting only the most significant genetic correlation, we performed the p values correction by the FDR method, using the software Seed-based d Mapping, formerly "Signed Differential Mapping" (SDM, version 6.22) [43]. Afterward, we selected those phenotypes with FDR < 0.05. Particularly, the aforementioned heritability and genetic correlation analyses [44] were performed using the 1000 Genomes Project (1KG) phase 3 European ancestry reference panel.

### Causal relationship analysis

In this step, we submitted the data of the most significant genetic correlations to the LCV package [45]. This package investigates the causal relationships, testing for the presence of a single latent variable connecting air pollutants exposure to respiratory diseases outcome. In particular, LCV was performed through R using 1KG phase 3 European ancestry reference LD panel. The estimated posterior genetic causality proportion (gcp) values obtained by this method indicate the percentage of causality between the phenotypes and the sign represents the causal direction. In this study a positive sign indicates a direct causal relationship from a pollutant to a respiratory disease. In order to select only the most significant causal relationships, we converted the log10(p) in non-logarithm p values and performed the p values correction by the FDR method. That was possible using the software Seed-based d Mapping, formerly "Signed Differential Mapping" (SDM), version 6.22 [43]. Afterwards we selected those phenotypes with FDR < 0.05.

### Clump analysis

Afterward, we focused on several representative pollutants with a significant causal relationship with respiratory diseases and extracted the list of their SNPs from each of their summary statistics. Subsequently, we submitted these lists to the PLINK v.1.90b package to remove the highly correlated SNPs and thus obtain the most descriptive SNPs. Indeed, variants may be in LD (i.e., physically close to each other along the chromosome, in areas defined as haplotype). For this purpose, we computed the LD-clumping analysis using the clumping procedure provided by PLINK v.1.90b [46] and using the 1KG as a reference. In this process, the algorithm generates clusters around the SNPs index (i.e., SNPs with the lowest p-value in the haplotype) with the following threshold values: Clump-p1: 1e-05 (significant threshold for index SNPs), Clump-p2: 0.01 (second significant threshold for clump SNPs), Clump-r2: 0.001 (pairwise correlation. LD threshold for clumping) and Clump-Kb: 10000 (physical distance threshold for clumping). Given the complex genetic architecture of pollutant-exposed subjects, we applied a condition to eliminate strongly associated variants. Thereby, we identified the variants most associated with the respective haplotype (i.e., SNP index) and consequently with the corresponding pollutants.

### Mapping analysis of genes and pathways

Finally, in order to investigate the genes effected by the aforementioned SNPs index, we submitted the lists of these variants to two R packages: EnsDb.Hsapiens.v79 (version 79, Johannes Rainer, Bolzano, Italy) [47] and Biomart (version 2.46.3, Steffen Durinck, Leuven-Heverlee, Belgium) [48, 49]. The lists of genes obtained by these packages were subsequently submitted to the Reactome software (version 77, Lincoln Stein, Toronto, Canada) for the pathway analysis [50]. Through this software, we identified several pathways whose mechanisms might have been influenced by the identified SNPs.

## Supporting information

S1 Table Significant 43 phenotypes based on heritability (h^2) with z-score > 4. An optimal value of Lambda is between 0.95 and 1.05. If Lambda RG is lower than 0.95, it could be due a bias (in an underpowered dataset). Otherwise, if it is greater than 1.05 it could be for two reasons: the dataset does not follow a normal distribution or due polygenicity. The case of polygenicity can be identified through the intercept. Indeed, a value of intercept close to 1 justifies the polygenicity hypothesis. SE: standard error; GC: genetic correlation.
(XLSX)

S2 Table Correlations between pollutants and respiratory disorders. The genetic correlation expresses two aspects: the percentage with which pollutants and respiratory disorders are correlated and the type of correlation (i.e., a positive value means correlation in the same way, a negative value means correlation in opposite way). GC (SE): genetic correlation standard error. FDR: false discovery rate p value.
(XLSX)

S3 Table List of phenotypes. It provides the complete list of the phenotypes analyzed in this work.
(XLSX)
